# In This Issue

**Published:** 2003

## ALCOHOL, OXIDATIVE STRESS, AND FREE RADICAL DAMAGE

When alcohol is broken down in the liver, some of the chemical reactions involved also result in the generation of reactive molecules called free radicals, including a group of oxygen-containing radicals called reactive oxygen species (ROS). The primary system that produces ROS during alcohol breakdown involves cytochrome P450 enzymes, which are particularly active after heavy alcohol consumption. Several other cellular systems that are influenced by alcohol also contribute to ROS formation. ROS can wreak havoc in the cells by damaging proteins, lipids, and DNA. Several mechanisms normally protect the body from the harmful actions of these ROS, but alcohol also interferes with these mechanisms. Excessive ROS production combined with impaired protective mechanisms results in a condition called oxidative stress, which has numerous deleterious consequences for the cells and tissues. In particular, oxidative stress may promote the development of alcoholic liver disease, as discussed by Drs. Defeng Wu and Arthur I. Cederbaum. (pp. 277–284)

## DANGEROUS BYPRODUCTS OF ALCOHOL BREAKDOWN—FOCUS ON ADDUCTS

Alcohol breakdown in the liver generates not only harmless products but also highly reactive and potentially harmful intermediates (i.e., acetaldehyde) and byproducts (e.g., reactive oxygen species). Drs. Dean J. Tuma and Carol A. Casey describe how all of these molecules can interact with other complex molecules in the cell, such as proteins, lipids, and DNA, to form hybrid molecules called adducts. These adducts can contribute to alcoholic liver disease—for example, by interfering with the functions of proteins such as hemoglobin and collagen or by triggering harmful immune responses. Evidence for the significance of adducts in the development of alcoholic liver disease comes from studies that demonstrate the presence of adducts in alcohol-consuming laboratory animals and humans and from findings that adducts are formed particularly in liver regions that are the first to show signs of alcoholic liver disease. (pp. 285–290)

## ENERGY AVAILABILITY AND ALCOHOL-RELATED LIVER PATHOLOGY

When liver cells break down alcohol, they use up more oxygen than usual, leading to oxygen deficits in regions of the liver that are exposed to low oxygen levels even under normal conditions. Adequate oxygen levels, however, are crucial for many cellular reactions, including the formation of a molecule called adenosine triphosphate (ATP), which provides the energy for many biochemical reactions in the cell. Most of the cell’s ATP is generated during the breakdown of the sugar glucose, particularly during a set of reactions known as mitochondrial oxidative phosphorylation. This process requires oxygen and cannot proceed efficiently in cells that are experiencing oxygen deficits. Drs. Carol C. Cunningham and Cynthia G. Van Horn review the mechanisms through which alcohol leads to oxygen deficits in the liver and impairs ATP generation. They also explore how these effects could contribute to cell damage and alcoholic liver disease. (pp. 291–299)

## ENDOTOXIN AND KUPFFER CELL ACTIVATION IN ALCOHOLIC LIVER DISEASE

One important pathway leading to alcoholic liver disease begins not with alcohol’s direct effects on the liver but with alcohol-induced changes in the wall of the intestine that allow a bacterial protein called endotoxin to enter the bloodstream. When endotoxin reaches the liver, it activates immune cells known as Kupffer cells, which then release signaling molecules (i.e., cytokines) and other harmful molecules, such as reactive oxygen species. These molecules in turn trigger responses in other liver cells that ultimately can induce liver damage. Dr. Michael D. Wheeler summarizes research findings elucidating various steps in this series of reactions, which involves a multitude of cells and molecules. This discussion focuses particularly on the events occurring when endotoxin interacts with and activates Kupffer cells, because detailed knowledge of these processes might lead to the development of novel therapeutic approaches that could prevent or ameliorate alcoholic liver disease. (pp. 300–306)

## CYTOKINES—CENTRAL FACTORS IN ALCOHOLIC LIVER DISEASE

For cells of different tissues or even within one tissue to coordinate their activities, chemical messengers must carry information from one cell to another. One class of these messengers are small molecules called cytokines, which are particularly important in mediating and coordinating inflammatory reactions (e.g., in response to an infection). According to Dr. Manuela G. Neuman, cytokines are essential for liver cells to function normally and respond to external influences, such as the presence of alcohol. Persistent cytokine secretion, however, can lead to chronic inflammation, which can result in hepatitis, fibrosis, and cirrhosis. Cytokines, particularly molecules called tumor necrosis factor alpha and transforming growth factor beta, also control a process known as programmed cell death, or apoptosis, which normally serves to eliminate damaged or unneeded cells. In the presence of alcohol, however, the activities of these cytokines in the liver are altered or enhanced, leading to excessive apoptosis and, consequently, destruction of liver tissue. (pp. 307–316)

## INTRACELLULAR PROTEOLYTIC SYSTEMS IN ALCOHOL-INDUCED TISSUE INJURY

Proteins play pivotal roles in almost all cellular functions, either by forming structural components of the cell or by acting as enzymes. For the cell to function properly, proteins also must be broken down in a coordinated manner to eliminate proteins that are damaged or are no longer needed. Protein breakdown, or proteolysis, is performed by several cellular systems, including cell structures called lysosomes, a set of reactions known as the ubiquitin–proteasome pathway, and enzymes called calpains. As Drs. Terrence M. Donohue, Jr., and Natalia A. Osna report, alcohol interferes with the activities of all three of these systems. As a result, proteins may accumulate in the liver, with potentially detrimental effects. Impaired proteolysis in the liver also can lead to inflammation and even liver cell death. These findings suggest that alcohol-induced disruption of proteolysis may be one of the pathways leading to alcoholic liver disease. (pp. 317–324)

## RESEARCH UPDATE: ANIMAL MODELS OF ALCOHOLIC LIVER DISEASE

Lacking a single animal model that simulates alcoholic liver disease (ALD) as it occurs in humans, researchers have used animal models to address specific questions about the disease. Drs. Amin A. Nanji and Samuel W. French discuss this body of research, with particular emphasis on the intragastric feeding model in rats, a method that allows tight experimental control of animals’ consumption of both alcohol and dietary nutrients. Research using the intragastric model has shed light on such issues as the mechanisms responsible for liver scarring in ALD and how ALD progression is affected by factors such as nutrition, oxygen deprivation (as occurs with sleep apnea or smoking), and gene regulation. (pp. 325–330)

